# Efficiency assessment of a novel automatic mosquito pupae sex separation system in support of area-wide male-based release strategies

**DOI:** 10.1038/s41598-024-58067-x

**Published:** 2024-04-22

**Authors:** W. Mamai, O. Bueno-Masso, T. Wallner, S. A. Nikièma, S. Meletiou, L. Deng, F. Balestrino, H. Yamada, J. Bouyer

**Affiliations:** 1 Insect Pest Control Laboratory, Joint FAO/IAEA Centre of Nuclear Techniques in Food and Agriculture, Vienna, Austria; 2 Institut de Recherche Agricole pour le Développement (IRAD), Yaoundé, Cameroun; 3 Institut de Recherche en Sciences de la Santé/Direction Régionale de l’Ouest (IRSS/DRO), Bobo-Dioulasso, Burkina Faso; 4 Cyprus University of Technology, Limassol, Cyprus; 5 Environmental Health Institute, National Environment Agency, Singapore, Singapore; 6 CIRAD, UMR ASTRE CIRAD-INRA “Animals, Health, Territories, Risks and Ecosystems”, Campus International de Baillarguet, 34398 Montpellier Cedex 05, France

**Keywords:** Biological techniques, Diseases

## Abstract

This study provides a comparative analysis of two state-of-the-art automatic mosquito pupae sex sorters currently available: the ORINNO and the WOLBAKI Biotech pupae sex separation systems, which both exploit the sexual size dimorphism of pupae. In *Aedes aegypti*, the WOLBAKI sex sorter and the ORINNO with a sieve mesh size of 1.050 mm achieved sex separation with female contamination rates below 1%, low pupae mortality rates and high male flight capacity. However, in *Ae. albopictus*, there was more variability, with female contamination rates above the 1% threshold and pupae mortality reaching 27% when using the ORINNO sorter. On the other hand, the WOLBAKI sorter achieved a male pupae recovery of 47.99 ± 8.81% and 50.91 ± 11.77% in *Ae. aegypti* and *Ae. albopictus,* respectively, while the ORINNO sorter with a smaller sieve size achieved male pupae recoveries of 38.08 ± 9.69% and 40.16 ± 2.73% in *Ae. aegypti* and *Ae. albopictus,* respectively. This study provides valuable information for researchers and practitioners in the field, assisting in the selection of the most suitable system for mosquito control, management and research programs depending on their specific requirements.

## Introduction

Effective mosquito control strategies are crucial in combating mosquito-borne diseases such as malaria, dengue fever, yellow fever, chikungunya, Zika, Japanese encephalitis, West Nile fever, Rift Valley fever and lymphatic filariasis. Most vector control interventions that rely heavily on insecticides are not sustainable owing to the increasing resistance of mosquitoes to the pesticides currently being used^1–4^. Due to this global challenge of insecticide resistance in most major vectors, along with the increase in the incidence of arbourviral diseases, the urgent need for complementary and more sustainable control methods that are less reliant on pesticides has become increasingly evident^5^.

Over the past decades, interest has turned to the sterile insect technique (SIT) as it is a powerful and environmental friendly method for controlling mosquito vector populations, capable of transmitting several pathogens causing human and animal diseases^6,7^. When SIT or the Incompatible Insect Technique (IIT) is considered in an area-wide integrated vector control program, the elimination or separation of females from males is an essential requirement, as it enables the release of sterilized male mosquitoes to suppress or eradicate target populations^8^.

In many insect species, sexually dimorphic characteristics, such as size, shape, color, or specific anatomical features, can be used to distinguish between males and females. While there have been some promising advancements in methods for mosquito sex separation, many of them are still in the development stage and are not yet widely used or available. These methods include the development of genetic sexing strains^9–13^, computer vision analysis^14^ and, recently, a two-step sorter to automate sex sorting pupae and adult *Aedes* mosquitoes^15^. One of the most common approaches for mosquito sex separation is size-based sorting, where female pupae are typically larger than males^16,17^. This is achieved through a series of sieves or filters with different mesh sizes or using glass plate separators. However, the use of metal sieving plates^18,19^ and the Fay-Morlan glass separator^20–23^ requires considerable time, staff, or have so far failed to eliminate more than ninety-nine percent of females to enable male-only releases for the SIT or other related applications. Sex separation remains a major challenge to be addressed to sustain large-scale SIT operations. Therefore, the demand for new tools that are both highly effective and scalable has reached a critical level, making their development and implementation more crucial than ever before.

Automatic mosquito sex sorting systems that can handle a large number of mosquitoes with minimal human intervention and achieve high accuracy hold immense potential to facilitate the efficient production and release of male mosquitoes on a large scale, offering a practical solution to enhance vector control efforts. The company WOLBAKI has capitalized on recent technological advancements and has successfully created an automated mosquito sex sorter (Model: WBK-P0001-V2) based on the manual Fay-Morlan sorter^24,25^. The WOLBAKI sex sorter has been fully evaluated in the laboratory on three mosquito species (*Ae. aegypti*, *Ae. albopictus* and *Culex quinquefasciatus*)^25^ and successfully tested in a field trial to suppress *Ae. albopictus* in an IIT program in China^24^. As part of ongoing efforts to overcome the challenge and improve the efficacy of sex separation, ORINNO Technology Pte Ltd., in collaboration with the National Environment Agency’s (NEA) Environmental Health Institute, Singapore, has designed and developed another automatic mosquito pupae sex separation system (Model No. PSS-220) to separate male and female mosquito pupae based on pupal size dimorphism. The system is currently being used in an SIT—IIT program in Singapore to separate the sexes of a Wolbachia-infected *Ae. aegypti* strain^26^. However, the efficacy of any sex-sorting device that relies on size differences can change with the mosquito species, the mass-rearing procedures used and the scale of the operation.

The current study aimed to assess the performance of the novel automatic mosquito pupae sex separation system developed by the ORINNO Technology Pte Ltd. We used a comparative approach against the automatic mosquito pupae separation system developed by the WOLBAKI. Therefore, we evaluated (i) its accuracy in effectively separating male and female pupae by comparing female and male contamination rates in male and female pupae batches, (ii) its impact on pupae mortality and male flight ability, and (iii) its applicability across two mosquito species, *Ae. albopictus* and *Ae. aegypti*. Moreover, we evaluated potential methods to reduce the female contamination rate arising from the presence of larvae in separated pupae.

## Results

### Female and male contamination rates

**Figure 1:**
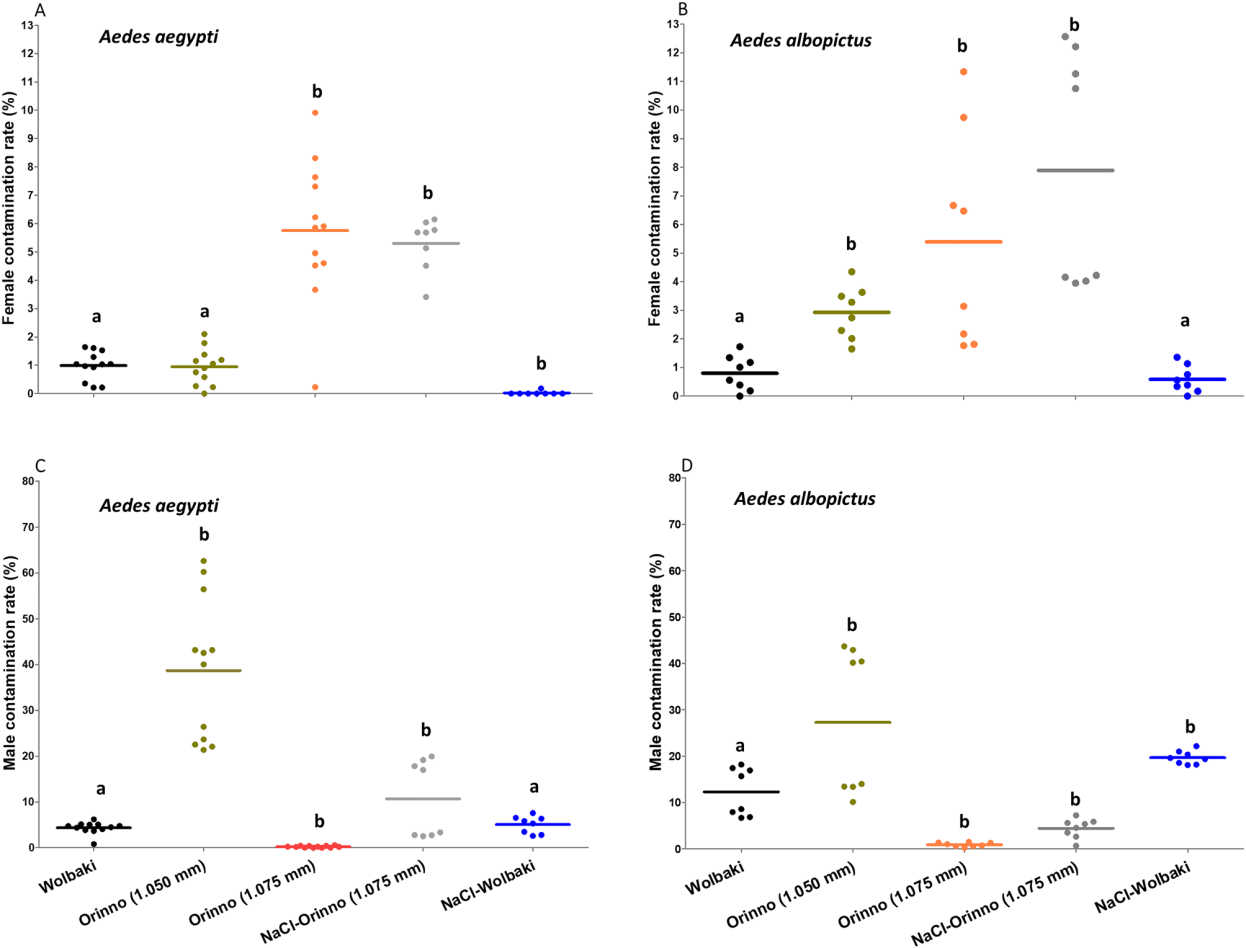
Female contamination percentage in male pupae fractions (**A,B**) and male contamination percentage in female pupae fractions (**C,D**). Data presented in the figure are expressed as mean ± SE. Values were compared to WOLBAKI alone as reference.

The percentages of female/male contamination for both species are presented in Fig. 1. Overall, the female/male contamination rates varied significantly across species and sorting methods used in this study.

In *Ae. aegypti*, among all the methods used, the combination 15% NaCl solution -WOLBAKI exhibited the lowest female contamination rate in the male pupae, with only 0.02 ± 0.02% (mean ± SE) of females being present in the sorted samples, which was significantly lower than WOLBAKI alone (0.98 ± 0.15, z = −3.74, p = 0.0002). WOLBAKI alone and ORINNO (1.050 mm mesh size) had 0.98 ± 0.15% and 0.94 ± 0.19% female contamination, respectively (less than the acceptable threshold of 1%), with no significant difference (z = 0.075, p = 0.94). On the other hand, ORINNO (1.075 mm mesh size) and 15% NaCl solution—ORINNO (1.075 mm mesh size) had the highest female contamination rates (5.76 ± 0.75% and 5.30 ± 0.35%, respectively).

Conversely, in *Ae. albopictus*, only sorting with the WOLBAKI sorter or with 15% NaCl solution—WOLBAKI had female contamination rates lower than the required 1% threshold (0.79 ± 0.22% and 0.59 ± 0.18%, respectively). The female contamination percentages were higher with ORINNO sorter, whether alone or combined with 15% NaCl solution treatment (Fig. 1B). We examined the level of male pupae loss in the female pupae fraction (male contamination rate). In this regard, ORINNO (1.050 mm mesh size) had the highest male contamination percentages (38.70 ± 4.66% and 27.28 ± 5.90% in *Ae. aegypti* and *Ae. albopictus,* respectively) in contrast to ORINNO (1.075 mm mesh size), which exhibited the lowest percentages of male contamination regardless of the species (0.22 ± 0.06% and 0.93 ± 0.17% in *Ae. aegypti* and *Ae. albopictus,* respectively; Fig. 1C, D). The WOLBAKI sorter exhibited 4.34 ± 0.39% and 27.28 ± 5.90% male contamination in *Ae. aegypti* and *Ae. albopictus,* respectively; Fig. 1C, D).

### Pupae recovery rates

From the male and female pupae estimated after one tilting/sorting event (40–44 h from the onset of pupation) and taking into account the female/male contamination percentages in each fraction, the partial male (actual percentage available for releases) and partial female pupae recovery rates were calculated, and the results are summarized in Table 1. In both species, the WOLBAKI sorter achieved the highest male pupae recovery percentages (47.99 ± 8.81% and 50.91 ± 11.77% in *Ae. aegypti* and *Ae. albopictus,* respectively), while the ORINNO sorter with the smaller sieve size decreased the percentage of male pupae recovered (38.08 ± 9.69% and 40.16 ± 2.73% in *Ae. aegypti* and *Ae. albopictus,* respectively).

**Table 1:** Percentages of male and female partial pupae recovery (mean ± se) in *Aedes aegypti* and *Aedes albopictus* using WOLBAKI and ORINNO automatic mosquito pupae sex sorters.

Species	Parameters	Sorting methods				
		WOLBAKI™	ORINNO Pte Ltd (1.050 mm)	ORINNO Pte Ltd (1.075 mm)	NaCl-ORINNO Pte Ltd (1.075 mm)	NaCl-WOLBAKI™
*Aedes aegypti*	Partial male recovery %	47.99 ± 8.81 a	38.08 ± 9.69 b	45.80 ± 6.76 a	43.01 ± 9.08 a	47.21 ± 10.72 a
	Partial female recovery %	20.06 ± 9.55 a	15.81 ± 10.59 a	13.30 ± 5.82 b	22.25 ± 1.57 a	22.85 ± 0.42 a
*Aedes albopictus*	Partial male recovery %	50.91 ± 11.77 a	40.16 ± 2.73 b	42.16 ± 2.36 a	42.28 ± 9.57 a	37.88 ± 19.10 b
	Partial female recovery %	13.19 ± 5.07 a	14.35 ± 3.15 a	6.50 ± 3.04 b	9.12 ± 6.79 a	7.43 ± 2.02 b

Values are expressed as the mean ± SE. Within a row, different letters with WOLBAKI indicate a statistically significant difference (p < 0.05).

### Assessing the fate of larvae contaminating the male pupae fraction

**Figure 2:**
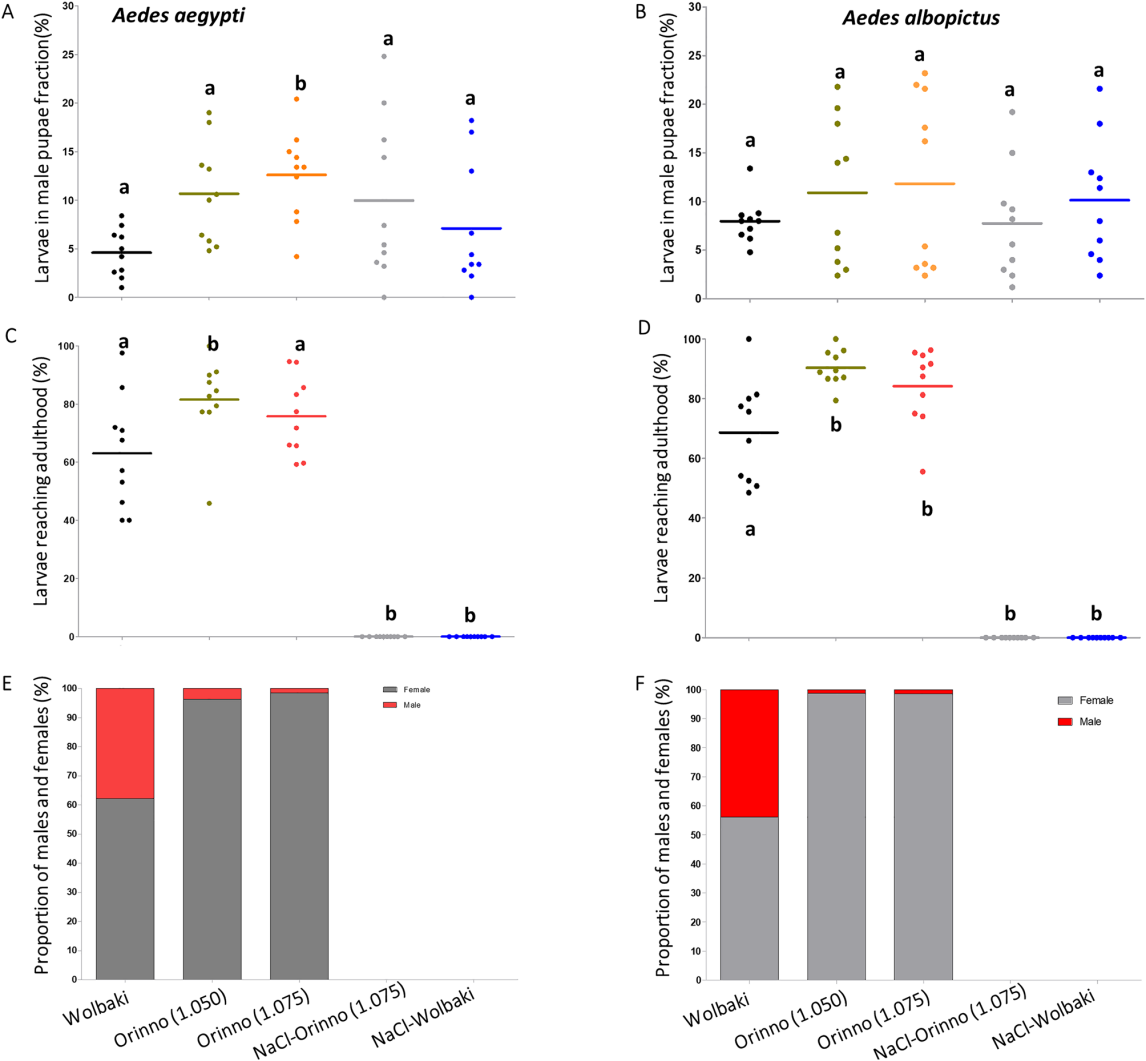
Fate of larvae contaminating the male pupae fraction after sex separation. Proportion of larvae in the male pupae fraction (**A,B**); proportion of contaminated larvae reaching adulthood (**C,D**); proportion of males and females from larvae that reached adulthood (**E,F**). Data presented in the figure are expressed as mean ± SE. Values were compared to WOLBAKI alone as reference.

After sorting following the abovementioned five different methods, larvae from each sample of approximately 500 pupae were removed and transferred to a separate burdorm cage and left for 4 days. Whatever the sorting methods, the level of larval contamination in the male pupae fraction varied from 0 to 24.8% (mean ± se; 8.98 ± 0.88%) in *Ae. aegypti* and from 1.2 to 23.2% (mean ± se; 9.72 ± 0.93%) in *Ae. albopictus.* The level of larval contamination varied between sorting methods in *Ae. aegypti* (Bartlett’s test, F = 2.9, p = 0.03, Fig. 2A), with the WOLBAKI sorter showing the lowest larval contamination but not a significant difference in *Ae. albopictus* Bartlett’s test, F = 0.75, p = 0.56, Fig. 2B). When the contaminating larvae were left for development for 4 days, 73.47 ± 3.20% and 81.06 ± 02.87% in *Ae. aegypti* and *Ae. albopictus,* respectively*,* reached adulthood (Fig. 2C, D). The WOLBAKI sorter exhibited a lower percentage of larvae reaching adulthood compared to the ORINNO sorter in both species. No contaminating larvae reached adulthood when treated with 15% NaCl solution for 1 h before sorting with both the WOLBAKI and the ORINNO sorters.

From those larvae that reached adulthood and both species combined, 59.19%, 97.53%, and 98.56% for WOLBAKI, ORINNO (1.050 mm mesh size) and ORINNO (1.075 mm mesh size), respectively, were female (Fig. 2E, F). If these larvae are included in the male pupae fraction, this can lead to an increase in the overall female contamination rate. We thus calculated the contamination of females that would have been obtained if the larvae had not been removed. The female contamination percentages were beyond 3% and reaching 15% in average. The results are summarised in the Table 2.

**Table 2:** Female contamination percentage in male pupae fractions calculated if contaminating larvae were left with pupae for emergence over 4 days in *Aedes aegypti* and *Aedes albopictus* using WOLBAKI and ORINNO automatic mosquito pupae sex sorters. Values are expressed as the mean ± SE.

		WOLBAKI™	ORINNO Pte Ltd (1.050 mm)	ORINNO Pte Ltd (1.075 mm)
*Aedes aegypti*	Female contamination if larvae were left for 4 days (%)	3.03 ± 0.15	8.86 ± 0.19	14.33 ± 0.76
*Aedes albopictus*	Female contamination if larvae were left for 4 days (%)	3.59 ± 0.22	11.74 ± 0.34	14.99 ± 1.42

### Male pupae mortality and flight ability

Male pupae mortality was low in *Ae. aegypti* after sex separation using either the ORINNO or the WOLBAKI automatic sorters (with an observed pupae mortality of 1.80 ± 0.55% and 1.20 ± 0.22% for the ORINNO and WOLBAKI sorters, respectively) and did not differ significantly between the two sorters (z = −0.775, p = 0.44). In *Ae. albopictus,* the male pupae mortality with the ORINNO sorter was high (27.1 ± 5.31%) and differed significantly from that with the WOLBAKI sorter (4.30 ± 0.74%) (z = −12.35, p < 2e−16).

**Figure 3:**
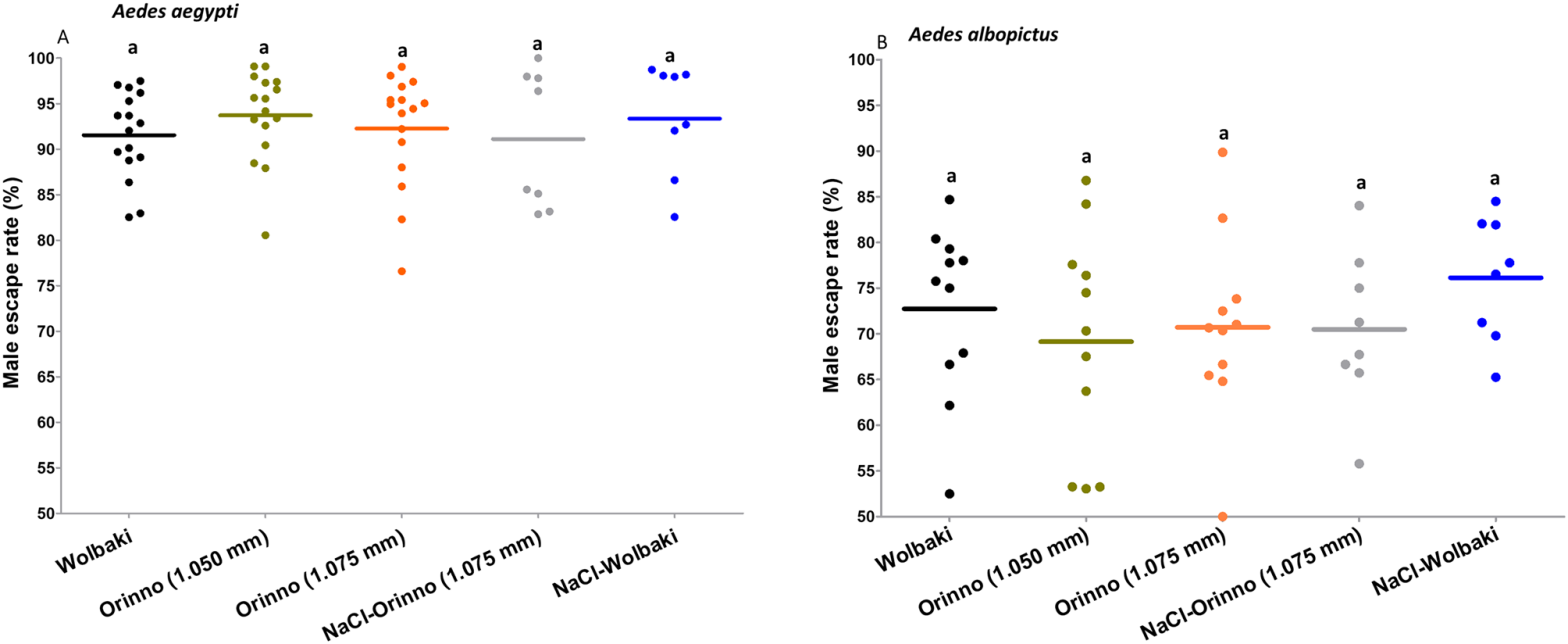
Male flight ability assessment in *Aedes aegypti* (**A**) and *Aedes albopictus* (**B**) after sex separation. Data presented in the figure are expressed as mean ± SE. Values were compared to WOLBAKI. There were no significant differences observed in the escape rates of males in other sorting methods when they are compared to the WOLBAKI alone as a reference point.

In adult males, the male flight ability did not differ significantly between the sorting methods (using the two automatic sorters and/or combined with 15% NaCl solution treatment) in either species (p > 0.05, Fig. 3). Male escape rates evaluated in adult males of 3–4 days of age were lower in *Ae. albopictus* than in *Ae. aegypti* regardless of the sorting method.

## Discussion

The accurate and efficient separation of males from females is crucial for the successful implementation of both SIT and other male release programs^27,28^. It is therefore essential to evaluate the suitability of a particular sex separation method for each target species individually before implementing it in large-scale programs. The present study compared the efficiency of two automated sorters for mosquito pupae sex separation on two mosquito species, *Ae. aegypti* and *Ae. albopictus*.

The separation efficiency of the two aforementioned sorters relies on the degree of pupae sexual size dimorphism within the mosquito species being sorted and the degree of size homogeneity within sexes. In *Ae. aegypti*, the WOLBAKI sorter and the ORINNO sorter with suitable sieve mesh size achieved less than a 1% female contamination rate with no detrimental effect on pupae mortality and quality of the resulting adults, supporting their suitability for use in SIT programs targeting *Ae. aegypti* mosquitoes. Finding these consistent and low rates of female contamination are reassuring and encouraging when compared to the results of Gunathilaka et al.^27^ who found a female contamination rate of 16% in *Ae. aegypti* using a manual Fay–Morlan glass separator. The ORINNO sorter (1.050 mm mesh size) gave good selectivity, but this resulted in the loss of 19.04% of the male production. Discarding such a significant number of males (38.70% males in female batches) with the ORINNO sorter to obtain the required low contamination rate could seriously affect the program in terms of the cost effectiveness of the mass rearing process. It is currently being used in a large-scale SIT-IIT program against *Ae. aegypti* in Singapore^26^. However, in *Ae. albopictus*, female contamination rates were higher with the ORINNO sorter, while the WOLBAKI sorter exhibited a female contamination rate of 0.80% and achieved the highest pupae recovery rates. This finding is consistent with that of Zeng et al.^24^ in China, where the WOLBAKI sorter was used successfully to suppress *Ae. albopictus* in an IIT program in an urban area. These results confirm the findings of Malfacini et al.^29^ who found a female contamination rate of 4.52 ± 0.29% with the sieve method and 0.71 ± 0.35% with the Fay-Morlan glass manual separator in *Ae. albopictus*. Although both sorters use the principle of pupal size dimorphism, they utilize different mechanisms. The low female contamination shown with the WOLBAKI sorter can be attributed to its greater flexibility and adaptability compared to the ORINNO sorter with fixed sieve mesh sizes. Indeed, the WOLBAKI sorter is subjected to calibration at the beginning or during the separation process to accommodate the desired purification level by omitting pupae of intermediate size to reduce the female contamination rate. However, this calibration depends on the operator skills and subjectivity to select the level of separation between stages, while with the ORINNO sorter, no skills are required for handling. Regarding time efficiency, it was observed that the WOLBAKI sorter’s efficiency depends on the density of the larvae-pupae mixture introduced into the sorting container. Conversely, the ORINNO sorter maintains a fixed sorting cycle time regardless of the density of the larvae-pupae mixture in the sorting container. In the conditions of the present study, it was observed that the estimated time required to sort the content of three rearing trays (initially containing 54,000 L1) was 11 min using the WOLBAKI automatic sorter and 13 min with the ORINNO sorter. In a previous comparative study, Gong et al.^25^ demonstrated that the male production capacity of the WOLBAKI automatic sex sorter increases by ~ 17-fold as compared to the manual sex separation using the Fay-Morlan sorter, which enable one person to produce 16 million males per week. However, further studies would be necessary to determine the optimal density for accurate sorting.

While the female contamination rate represents an important parameter to be determined in regard to considering SIT programs, minimizing pupae mortality during sorting procedures is crucial to maintain high-density releases. Our results showed that both sorting methods have relatively low pupae mortality rates in *Ae. aegypti*, indicating their effectiveness in preserving the viability of pupae during the sorting process. However, the increase in pupae mortality observed in *Ae. albopictus* being sorted with the ORINNO sorter may be attributed to the sensitivity of this species to the mechanical stress caused by friction through sieving plates, resulting in damage to the pupae, while the WOLBAKI sorter may be less stressful and preserve the integrity of the mosquito pupae. In other comparative studies, *Ae. albopictus* was found to be more susceptible than *Ae. aegypti* to various factors, such as increasing water hardness/electrical conductivity^30^, underlining differences between these species. In Singapore’s large-scale SIT-IIT program against *Ae. aegypti* mosquitoes, a more sophisticated machine vision system is being used to analyze pupae size distribution prior to the sex sorting process. This allows a more accurate adaptive sieve size selection for ORINNO sorter to overcome pupal size variation between each production batch. Combined with an optimized rearing protocol, stringent rearing environmental control and 15% NaCl solution treatment, the team was able to achieve stable low female contamination (< 0.1%) in large-scale production.

Both sorters demonstrated variations in their performance of sex separating *Aedes* mosquito pupae across the two mosquito species tested with *Ae. aegypti,* showing better performance in terms of female contamination, recovery rates and male flight ability. Each species/strain may have distinct inherent characteristics that could impact the effectiveness of sex separation methods. Moreover, factors such as temperature, nutrition, and larval density can influence the development of male and female mosquitoes differently between species. Compared to other sorting methods, such as the manual Fay-Morlan glass sorter and sieving plates, one significant advantage of these automatic sorters is their ability to reduce workload and the risk of human errors that may occur during manual sorting^25,31^. While the initial investment can be substantial, it can lead to significant long-term cost savings: lower labor costs, minimized human errors, and optimized resource allocation. For SIT programs, male quality, efficiency and minimal female contamination are critical. Although each method has advantages and disadvantages, the choice depends on your specific needs, budget constraints, and the scale of your mosquito control operation. For small-scale operations or pilot projects, manual sorting might be cost-effective initially if the primary concern is cost, but as the scale increases and high accuracy is crucial, automated systems become more cost-effective in the long term. For the two automatic sorters used in this study, the current costs are €39,000 and €15,000 for the WOLBAKI and ORINNO respectively.

One of the challenges in mosquito sex separation is the potential for larval contamination, where female larvae mistakenly end up in the batch of male mosquitoes. This can lead to unintended increased release of female mosquitoes. Depending on the automatic sorter, 4 to 12% of larvae can be found in the male pupae fraction. If they are not removed, the presence of these larvae could increase female contamination. With 15% NaCl solution treatment for one hour, as demonstrated by Deng Lu (personal communication), none of these undesired larvae reached adulthood.

## Conclusion

The efficiency of the ORINNO automatic sex sorter was evaluated in comparison with that of the WOLBAKI automatic sex sorter. Overall, both automatic sorters had positive results, enabling the handling of larger volumes. This represents a significant step towards SIT large-scale operations. In *Ae. aegypti*, both automatic sorters appear to be viable options for mosquito sex sorting, with relatively low pupae mortality rates observed in this study. For instance, and under the conditions of the present study, the WOLBAKI sorter may be preferred (particularly for *Ae. albopictus*) as it reduces the number of female mosquitoes in the male fraction to a minimum while also minimising the loss of male mosquitoes. However, the selection of a specific technology/method will depend on the specific mosquito sorting needs or application, taking into account factors such as the species, cost and ease of operation. It is important to note that no method is completely foolproof, and there is always a possibility of some degree of contamination and room for improvement. Therefore, by combining automated sorting technologies with other methods, such as 15% NaCl solution treatment, the chances of contamination can be significantly reduced, ensuring the release of predominantly male mosquitoes for effective population control. Despite their numerous advantages, automatic sorters may also face challenges when the rearing procedures are not standardized (synchronisation of egg hatching, larval density, larval feeding, environmental conditions). These findings contribute to the understanding of sex separation methods and provide valuable information for researchers and practitioners in the field, assisting in the selection of suitable methods for accurate and efficient mosquito sex sorting.

## Material and methods

### The ORINNO automatic mosquito pupae sex sorter and its operating principle

**Figure 4:**
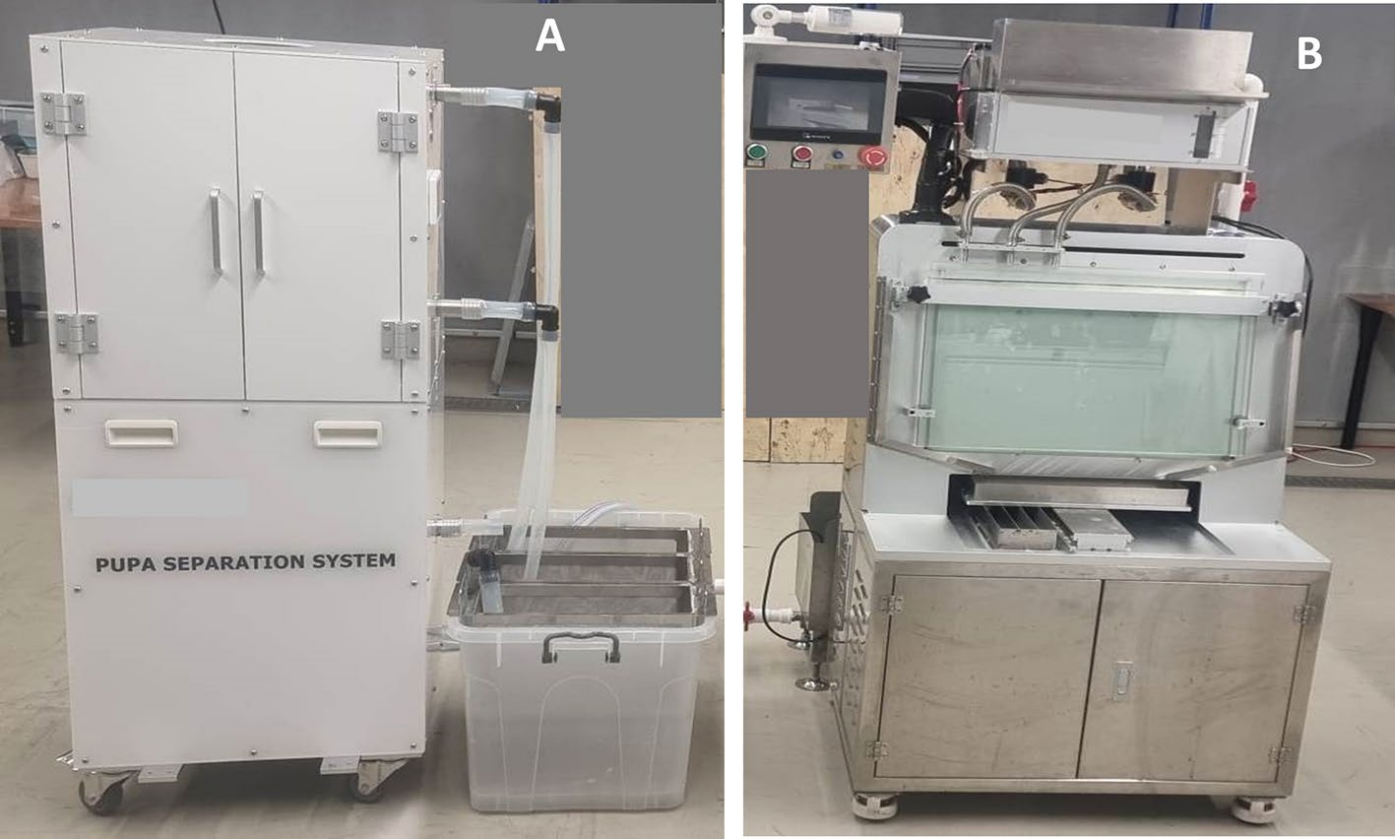
The ORINNO technology automatic pupae sex sorter (**A**) and the WOLBAKI automatic pupae sex sorter (**B**).

The ORINNO automatic mosquito pupae sex sorter is illustrated in Fig. 4A. Details on prices, specifications and manuals can be obtained from the manufacturer^32^. This sorter is designed for the separation of the developmental stages and sexes of mosquitoes. It is based on the principle that at the pupal stage, female mosquitoes are typically larger than their male counterparts, allowing for differentiation based on size. The system consists of a series of two sieves of different rectangular mesh sizes and is made of metal/aluminum material (Fig. 4A). After tilting the rack^31^, the mixture of pupae and larvae is introduced into the sorting system. The larger female pupae become trapped in the upper sieve with a smaller size, while the smaller male pupae and larvae can pass through and proceed to the lower sieve, which in turn retains male pupae, whereas larvae pass through. The mesh sizes of the sieving plate devices were chosen and constructed based on average male and female pupae cephalothorax size.

At the end of the sieving process, batches of male and female pupae and larvae are flushed with water and collected separately in corresponding containers.

The WOLBAKI automatic pupae sex sorter (Fig. 4B) design, structure and operating principle are described in Gong et al.^25^. Details on prices, specifications and manuals can be obtained from the manufacturer^33^. It also exploits the principle of size difference between male and female pupae by using two glass plates.

### Mosquito colonies and maintenance

*Aedes aegypti* (Juazeiro, Brazil strain) and *Ae. albopictus* (Rimini, Italy strain) were used in this study. Both species have been maintained at the Insect Pest Control Laboratory (IPCL), Seibersdorf, Austria since 2012 and 2018, respectively, following Food and Agriculture Organization (FAO)/International Atomic Energy Agency (IAEA) routine colony maintenance procedures^34^ and in mass rearing conditions^35^.

### Comparison testing of the ORINNO automatic sex sorter against the WOLBAKI sex sorter

For both species, 18,000 first instars per tray were fed with a 4% IAEA liquid diet following *Aedes* mass rearing procedures outlined in FAO/IAEA^35^ with a modified feeding regime implemented as follows: 300 mL on day 1 (20 h after egg hatching), 300 mL on day 4, 200 mL on day 5 and 300 mL on day 6. Trays were then tilted from the larval rearing rack, and their contents underwent a process of sex separation. During preliminary analysis, the presence of larvae in the male pupae fraction was observed after sorting, which could contribute to unpredictable female contamination rates (4 to 24%) higher than the acceptable rate of 1% for field releases^36^. To address larval contamination in pupae batches after sex sorting, a preliminary treatment of 15% NaCl solution was used to selectively kill larvae before sex separation procedures, as recommended by Deng Lu (personal communication). Our results also showed that 15% NaCl solution treatment for 1 h had no impact on pupal mortality, emergence and male flight ability (data not shown). For the subsequent experiments, the trays were tilted on day 7 (40–44 h after first pupation). Briefly, for each mosquito species, larvae for 15 mass-rearing trays were reared at the same time. For each of the 5 following sorting methods, the contents of three mass-rearing trays (18,000 initial larvae × 3 = 54,000) were tilted and mixed together. (a) ORINNO automatic sorter using a 1.050 mm mesh sieve, (b) ORINNO automatic sorter using a 1.075 mm mesh sieve, (c) WOLBAKI automatic sorter, (d) treatment with a 15% NaCl solution for 1 h followed by an ORINNO sorter using a 1.075 mm mesh sieve, and (e) treatment with a 15% NaCl solution for 1 h followed by a WOLBAKI automatic sorter. For each automatic sorter, the time spent for sorting the content of three trays was recorded to estimate time efficiency.

After the sorting process, batches of male and female pupae for each sorting method were estimated volumetrically using a modified tube^35^. After pupae estimation, four aliquots of approximately 500 pupae per sex and per treatment were randomly taken to measure the different parameters. When larval contamination is present in the male pupae fraction, it can increase the level of female contamination in the final samples. In all aliquots, the larvae (L4 and lower stages) were manually removed. To check for the fate of larvae contaminating the male pupae fractions and their contribution to the increase in female contamination, larvae from male pupae samples were counted and transferred to separate cages for 4 days. This observation period was selected based on the duration time adopted to remove the male pupae cups from emergence cages before adult mosquito irradiation procedures. They were allowed to pupate and emerge, and the resulting adults were counted by sex. Pupae samples in bowls with 75 mL of reverse osmosis water were also placed into emergence cages (15 × 15 × 15 cm^3^, BugDorm, Taichung, Taiwan) for 4 days. After emergence and when all adults had died, they were visually separated by sex (or when in doubt verified under a stereo-microscope) to determine female/male contamination percentages. The recovery percentages of male pupae or female pupae were determined taking into account the respective percentage of females or males found in the batches of male and female pupae. Additionally, approximately 500 male pupae (for each method) were randomly transferred into 15 × 15 × 15 cm^3^ emergence cages (BugDorm, Taichung, Taiwan) containing 10% sugar solution. After complete emergence, male flight ability tests (4 replicates of 100 males for each method) were performed following the method developed by FAO/IAEA^37,38^ on 3- to 4-day-old adult males. The entire experiment was repeated three times.

### Assessing the effect of using automatic pupae separation systems on pupal mortality

To investigate whether there was any detrimental effect of the different automatic pupae sex sorters on pupal mortality, five batches of 100 male pupae were manually counted after separation by both ORINNO and WOLBAKI sorters. Pupae were placed into bowls with 50 mL of reverse osmosis water and transferred into 15 × 15 × 15 cm^3^ cages. Pupal mortality was determined by manually counting the dead pupae every day for three consecutive days. The mortality rate was calculated by dividing the total dead pupae by the initial number of pupae.

### Statistical analysis

In this experiment, the female (or male) contamination rate was calculated for each sample (a batch of around 500 pupae). It was determined by dividing the number of adult males (or females) found in the batch after emergence by the total number of emerged adults from the same batch. After estimating the total number of male and female pupae volumetrically, the male total pupation rate was determined. This rate is calculated as the total number of male pupae formed divided by the initial number of male larvae, considering equal numbers of males and females in the initial larvae count. Finally, from male (or female) pupation percentages, male (or female) pupae recovery rates were calculated by removing the percentage of females (or males) in the male (or female) pupae count. Descriptive statistics and graphical representations were generated using GraphPad Prism 5.0 software. The R Software version 4.3.0 (R Development Core Team 2008, http://www.R-project.org/) along with RStudio (RStudio, Inc. Boston, MA, USA, 2016) were used to compare the data. Binomial generalized linear mixed models fit by maximum likelihood (Laplace Approximation) were used to examine all data with female and male contamination rates, female and male recovery rates and male flight ability as response variables. The sorting method was considered as a fixed effect, while the replicate and experiment were treated as random effects. To ensure the validity of the full models, Bolker's function was employed to detect overdispersion.

## Data Availability

All data generated or analyzed during this study are included in this published article.
